# Grazing-incidence scattering surveyed: towards reference methods for alignment and calibration

**DOI:** 10.1107/S1600576726005741

**Published:** 2026-07-24

**Authors:** Anja F. Hörmann, Daniel M. Balazs, Ingo Breßler, Sumea Klokic, Melika Moradi, Eduardo Solano, Annika Stellhorn, Brian R. Pauw

**Affiliations:** a Bundesanstalt für Materialforschung und -prüfung, Unter den Eichen 87, 12205 Berlin, Germany; b Institute of Science and Technology Austria, Am Campus 1, 3400 Klosterneuburg, Austria; c Graz University of Technology, Stremayrgasse 9, 8010 Graz, Austria; d ALBA Synchrotron, Carrer de la Llum 2-26, 08290 Cerdanyola del Vallès, Barcelona, Spain; e European Spallation Source ERIC Box 176 SE-221 00 Lund Sweden; Argonne National Laboratory, USA

**Keywords:** grazing incidence, reference methods, calibration, standardization, community

## Abstract

Grazing-incidence small-angle scattering (GISAS) is a relatively young technique with important applications in thin-film technology and untapped potential when it comes to 2D analysis on an absolute intensity scale. Approaching standardization and reference methods early is foundational for reproducibility and comparability across laboratories and reduction of systematic error sources. It underpins trust in data obtained and accelerates innovation by ensuring that scientists work from a common methodological baseline. Accordingly, obtaining reproducible results from different GISAS instruments requires an agreement on how measurements are performed, instruments calibrated and terms defined. To pave the way for standardization and reference methods, we surveyed GISAS practitioners on what comes before an experiment: hardware, software, sample alignment and instrument calibration. Twenty-two questions were designed to elucidate the state of the art, which can be used for the development of reference methods. Our data on 27 instruments provide the basis for standardization. With very few exceptions, we found laboratories prepared to implement future reference methods, but no consensus emerges naturally for sample alignment and instrument calibration. We, that is the GISAS community, are thus in a position to embark on the journey of standardization.

## Abstract 2

With the recent gain in popularity of grazing-incidence instruments, harmonization is needed to establish intercomparability. This review of existing instruments and methods highlights the diversity of approaches in the community and is a first step towards reference methods.

## 1. Introduction

Grazing-incidence (GI) scattering and reflectometry are techniques developed to study thin films on flat surfaces. Common objectives of GI scattering experiments are lattice parameters (Smilgies, 2025 ▸) for periodic structures on the nanoscale (Wernecke *et al.*, 2012 ▸; Vegso *et al.*, 2012 ▸; Frömsdorf *et al.*, 2006 ▸) or molecular scale (Kuhn *et al.*, 2025 ▸), and nanostructure orientation (Blanton *et al.*, 2000 ▸; DeLongchamp *et al.*, 2010 ▸). Reflectometry (Daillant & Gibaud, 2009 ▸) experiments (a different method that can be carried out on the same equipment) yield scattering length density profiles of thin films. While these profiles are informative for the analysis of GI measurements, critical angles and waveguide resonances are essential and can be obtained from a partial reflectometry experiment (Smilgies, 2021 ▸).

GI scattering began to grow as a field in the 1990s (Renaud *et al.*, 2009 ▸; Köhler *et al.*, 2026 ▸) and continues to attract researchers’ attention, as shown by publication activity (supporting information, Fig. S1), development of specialized software [see Hexemer & Müller-Buschbaum’s (2015 ▸) review, Pospelov *et al.* (2020 ▸), Reus *et al.* (2024 ▸) and Abukaev *et al.* (2026 ▸)] and scientific events. There were three international grazing-incidence small-angle scattering (GISAS) conferences fully dedicated to the topic up to 2015 (Dacombe, 2017 ▸), there have been several workshops [with year of latest event in parentheses: GISAS schools in Bayreuth/Munich, https://www.herzig.uni-bayreuth.de/en/Summer-School/gisas-2018/index.html (2018 ▸), Born­Again schools, https://bornagainproject.org/news/ba-school-2/ (2018 ▸), GISAXS workshops at DESY, https://indico.desy.de/event/48475/ (2025 ▸) (IUCr Commission on Small-Angle Scattering, 2026 ▸) and GISANS – Advancing data reduction and analysis, https://workshops.ill.fr/event/573/ (2026 ▸)], and conference contributions on GI scattering are now commonplace, *e.g.* at surface X-ray and neutron scattering (SXNS) and small-angle scattering (SAS) conferences.

Obtaining reproducible results from different GI scattering and reflectometry instruments requires an agreement on how the measurements are performed, instruments calibrated and terms defined. Although discussion of alignment and calibration has increased in recent years (Smilgies, 2021 ▸; Holzer *et al.*, 2022 ▸; Steele *et al.*, 2023 ▸; Werzer *et al.*, 2024 ▸; Tortorici & Rogers, 2025 ▸), it remains an open question whether the GI community has converged on a methodology. Therefore, we designed a survey around this question, the result of which can serve as a starting point for standardization and reference methods for GI scattering. In contrast to reflectometry [which emerged in earlier decades and has undergone standardization (International Organization for Standardization, 2020 ▸) to some extent], GI is a good candidate for this effort since it emerged in the 1980s (Vineyard, 1982 ▸), has a degree of variability due to the necessary alignment of every sample (Holzer *et al.*, 2022 ▸) and, at the time of writing, requires considerable effort for analysis. This paper and the underlying questionnaire focus on the preparation of a GI experiment: hardware, software, sample alignment and calibration.

## 2. Survey methodology

### 2.1. Questionnaire design

The questionnaire attempts to strike a balance between attention to detail and consideration of respondents’ time. The number of questions was limited to 22 (supporting information, Table S1) with the later addition of an optional field for a contact e-mail. The scope of the form was limited to hardware, software, alignment and calibration routines, with two main goals:

(i) To ensure that a standard developed on this basis can be implemented by as many instruments as possible.

(ii) To learn about the best methods by allowing respondents to share bespoke solutions they have developed.

We employed a mixture of multiple-choice, short free-form text and optional long free-form text. Two github gists were set up to allow respondents to contribute code samples, sample holder images and comments. The decision to allow, but not require, more in-depth responses was made deliberately to encourage participants to respond, but this meant that the reason behind the answers often remained unknown.

### 2.2. Data collection

The survey was communicated/advertised via domain-relevant conferences, the sa_scat@iucr.org mailing list and personal e-mail communication between July 2024 and March 2025. On 18 July 2024 at SXNS17, the survey was introduced and access shared via a QR code in session (attendance: a few dozen). Later that day an announcement e-mail followed to the sa_scat@iucr.org mailing list (with 363 subscribers as of 17 April 2025). Further responses were gathered by word of mouth as well as through individual encouragement to (beamline) scientists by the authors, followed by a second and third oral presentation at canSAS and SAS2024 (Taipei, Taiwan; attendance: a few dozen). Responses received until 4 March 2025 are considered for the present communication. Since our own GISAXS setup in the laboratory remains under development we did not complete the survey ourselves.

### 2.3. Response

Twenty-seven (27) responses were received, which include four neutron-based instruments and five using laboratory X-ray sources (Table 1 ▸). Respondents overwhelmingly work in Europe and North America (supporting information, Table S2). We make an attempt at quantifying the number of instruments per category below, but the distribution over continents already indicates blind spots in our sample. Nevertheless, if there is disagreement in our data we can say that the community has not converged on a single methodology.

#### 2.3.1. Estimation of the response potential

Quantification of the global potential for responses is difficult: first of all, the number of laboratory X-ray instruments is unknown (a user can build their own); second, there are associated techniques like GI diffraction, near-surface small-angle neutron scattering and GI total X-ray scattering sharing similar challenges; and, finally, instruments under construction are of relevance as well when the aim is harmonizing the methodology.

For our three categories of instruments (neutron, synchrotron, laboratory X-ray), we have the following approximate numbers: at least 11 with GI small-angle neutron scattering (GISANS) capabilities^1^ with none dedicated to GISANS (Köhler *et al.*, 2026 ▸), 44 synchrotron GI small-angle X-ray scattering (GISAXS) instruments^2^ and at least 57 laboratory X-ray scattering instruments with GI options.^3^ The fact that Xenocs has sold around 150 GISAXS stages overall (personal communication) indicates the upper bound is much higher, but acquisition of a GISAXS stage does not imply active use of the technique.

Scientific publications in recent years are a better proxy for active use of a technique than a mention on the instrument website. The OpenAlex scientific knowledge graph (Priem *et al.*, 2022 ▸) lists 1703 articles from 2020 to 2025 for the search terms GISAXS, GIWAXS, GISANS and GIXRD^4^ (supporting information, Table S4) (where GIWAXS is GI wide-angle X-ray scattering and GIXRD is GI X-ray diffraction). This corresponds to 283 articles per year on average. If we estimate that an ‘active’ instrument leads to of the order of 10 of these publications each year, we arrive at of the order of 30 instruments worldwide with a wide range of plausible values, 30 to 60, for 5 to 10 publications per instrument and year as assumptions. This estimate is lower than the manually counted results above (112 instruments overall) but consistent if many instruments only lead to the occasional GI publication.

Using the latter estimate (30 to 60 instruments), we have reached 48% to 95% of instruments with our survey. This fact allows us to consider our results relevant even though methods of drawing representative samples could not be applied to this problem.

## 3. Results

### 3.1. Hardware and software

**Figure 1:**
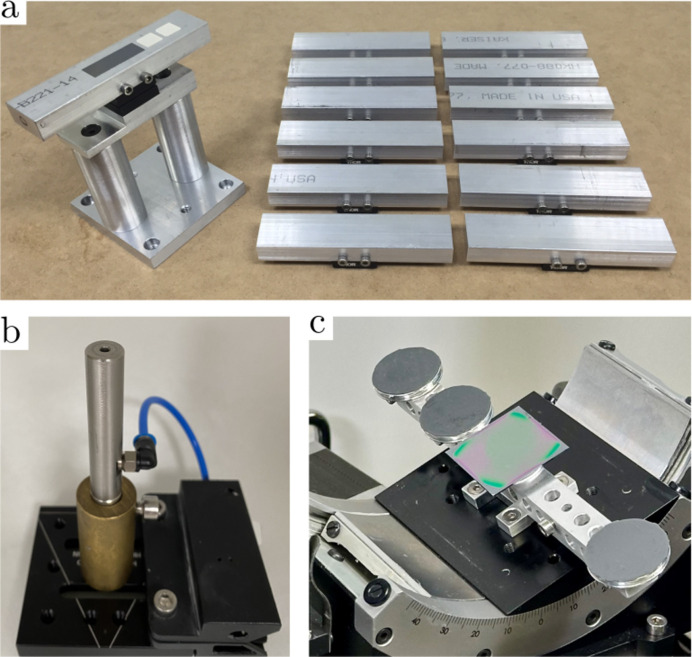
Examples of sample holders with different mechanisms for holding samples in place. (*a*) Gravity: bars for multiple samples. The typical sample shown on the left is shorter than the bar width, such that spilled beam is absorbed by the aluminium. Photograph courtesy of Ruipeng Li (BNL). (*b*) Vacuum: cylindrical support for one sample with a 3 mm hole connected to a vacuum pump. Photograph by Eduardo Solano (ALBA). (*c*) van der Waals forces: holder for up to five samples which are held in place by silicone grease with a sample of typical size (30 mm × 30 mm). Photograph by Brian R. Pauw (BAM).

Specialized hardware and software form the necessary backbone of experimental science, but we do not consider them parts of the methodology to be standardized (see examples of sample holders in Fig. 1 ▸). However, hardware and software are of relevance for our first goal: to ensure that as many instruments as possible can implement a standard developed on this basis.

**Figure 2:**
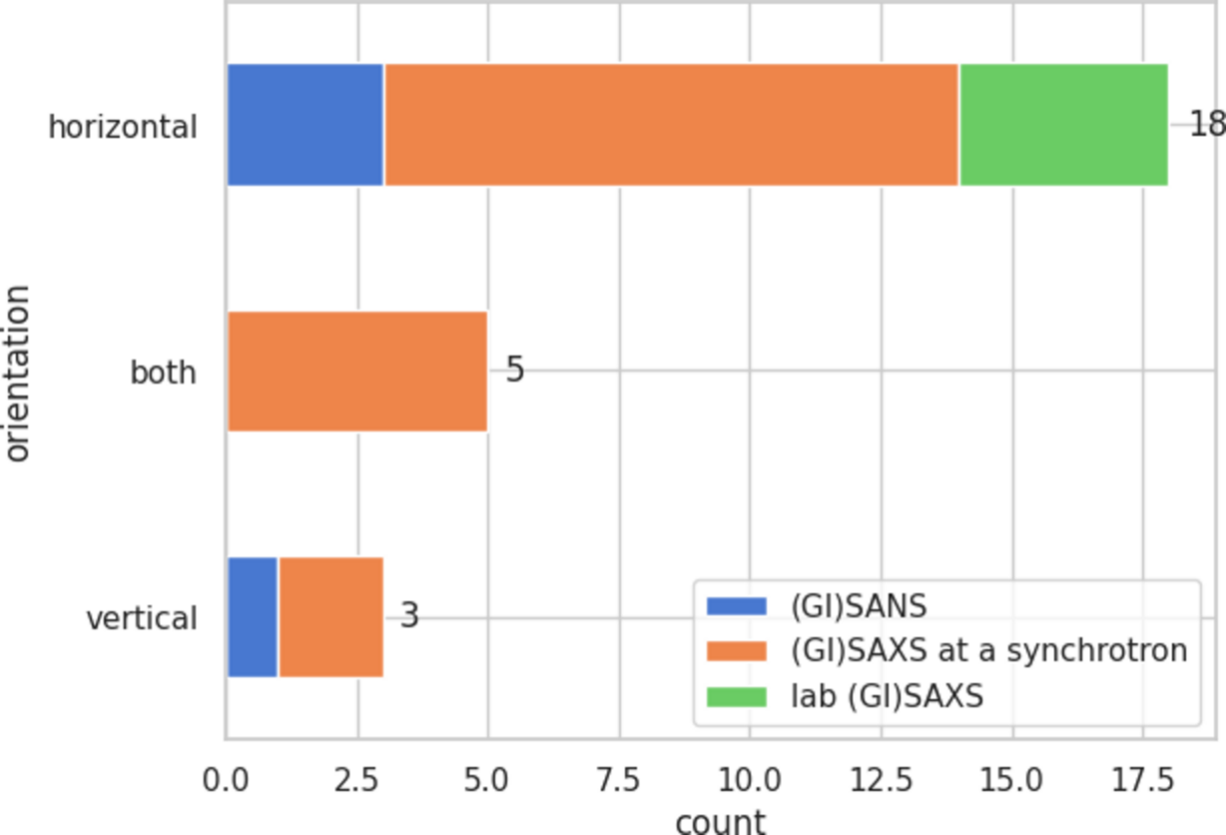
Typical sample orientation.

As shown in Fig. 2 ▸, the majority of instruments in our dataset use a horizontal sample orientation (that is, sample normal parallel to the *y* axis in the NeXuS coordinate system at incident angle zero). This prevalence of horizontal sample orientation may reflect the difference in horizontal and vertical emittance at synchrotron sources (Thompson, 2009 ▸).

In addition, the horizontal orientation is advantageous for samples influenced by gravity, although for measuring liquid surfaces the ability to pitch the beam is required as well.

**Figure 3:**
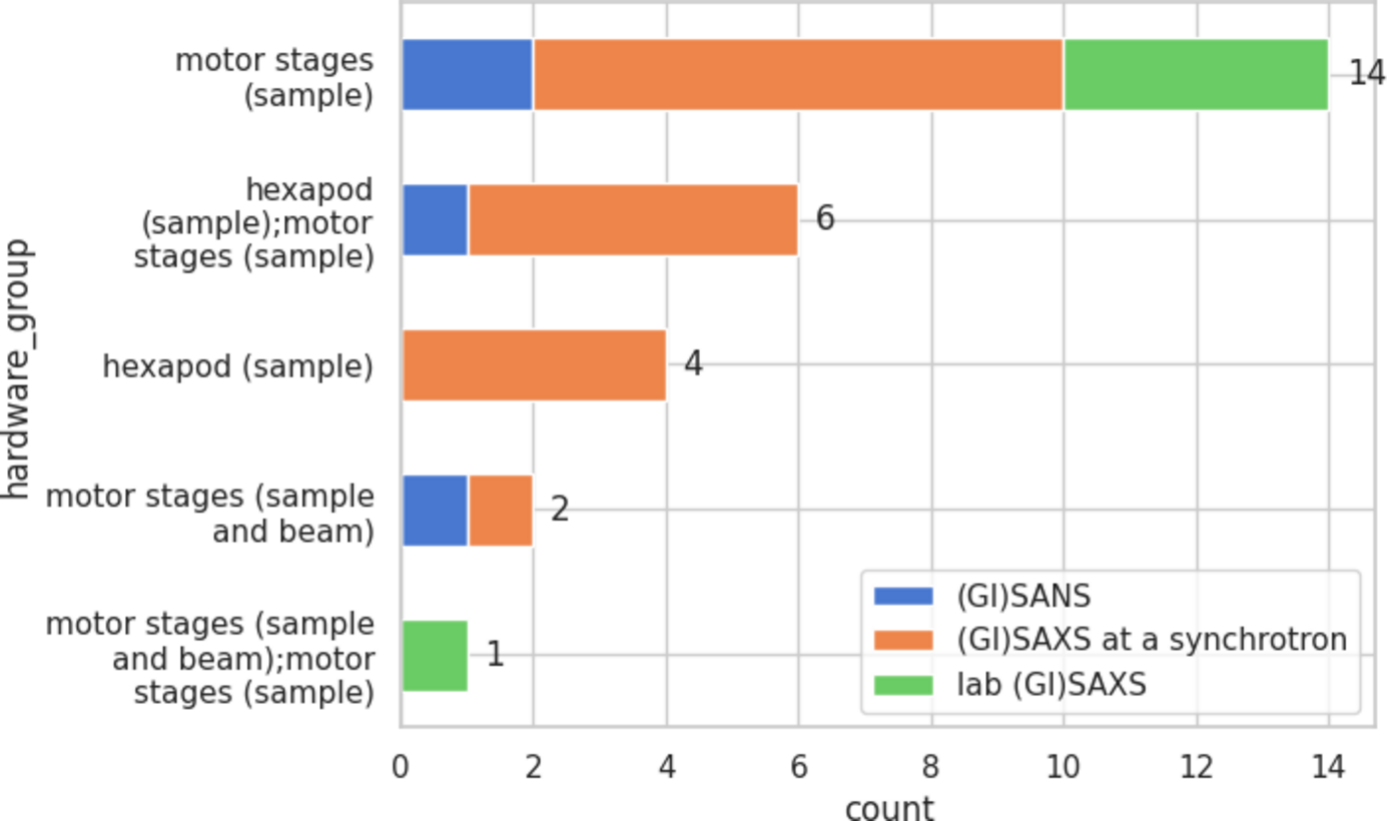
Combinations of hardware available to achieve sample alignment and control the incident angle. The majority of respondents indicate the use of individual motor stages for the translatory and rotatory degrees of freedom, with hexapods available at 37% of instruments (10/27). Multiple answers were allowed for this multiple-choice question.

**Figure 4:**
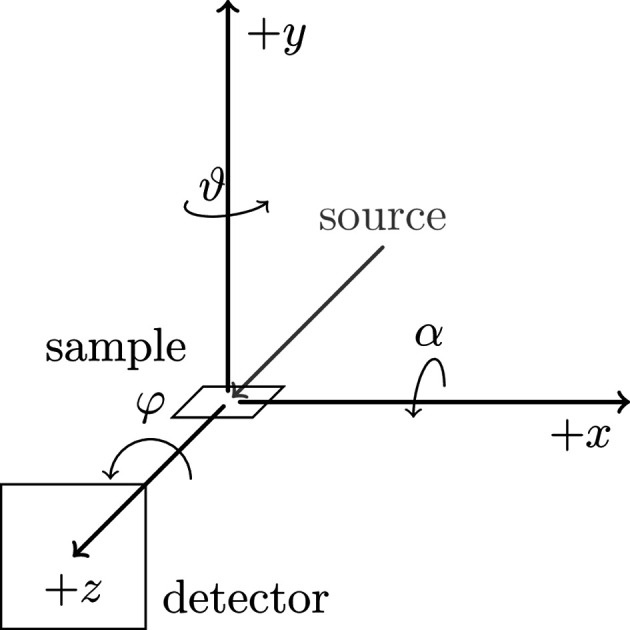
NeXuS coordinate system labeled with angle symbols as used in this work. See also Table 2 (Appendix *A*[App appa]).

The availability of different categories of hardware used to influence the relative orientations of the sample and the beam is visualized in Fig. 3 ▸. Most instruments employ motor stages for this purpose, with hexapods available at approximately a third (10 out of 27). Only three respondents indicated the ability to tilt the beam (Fig. 3 ▸). In practice, we expect that there are abstractions in place at each instrument which translate hardware motion to, for example, NeXuS coordinates (Fig. 4 ▸).

To coordinate and control measurement tasks, the vast majority of respondents have a scriptable software interface (25/27) where routines *e.g.* for alignment are under scientists’ control (25/27). The ability to automate and adapt routines is, for standardization purposes, more important than any specific software used (supporting information, Fig. S3). To conclude, the hardware and software layers available are adjustable to accommodate standardized alignment and calibration tasks.

### 3.2. Sample alignment

The term ‘sample alignment’ refers to the process of determining the position of the sample (and/or beam) where the beam propagation vector coincides with the surface. Roll alignment (φ in the horizontal sample orientation, see Fig. 4 ▸), that is ensuring that the sample normal coincides with one of the axes of the detector pixel array, is not mentioned by all respondents; it is therefore unclear whether this step is considered optional by some (for laboratory instruments, vendor product portfolio and pricing may restrict scientists’ options).

**Figure 5:**
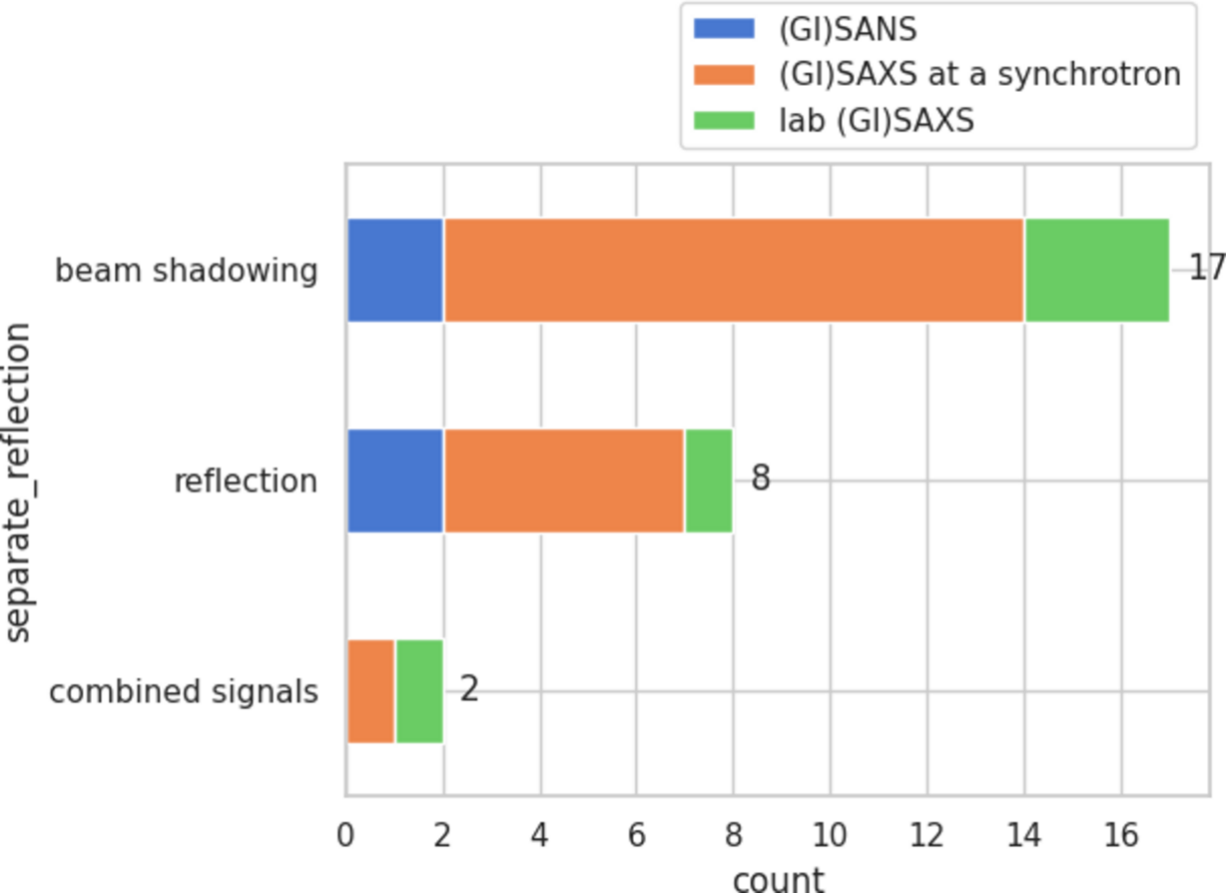
Signals available to quantify shadowing of the beam. Eight respondents report relying solely on the specular reflection for alignment purposes.

**Figure 6:**
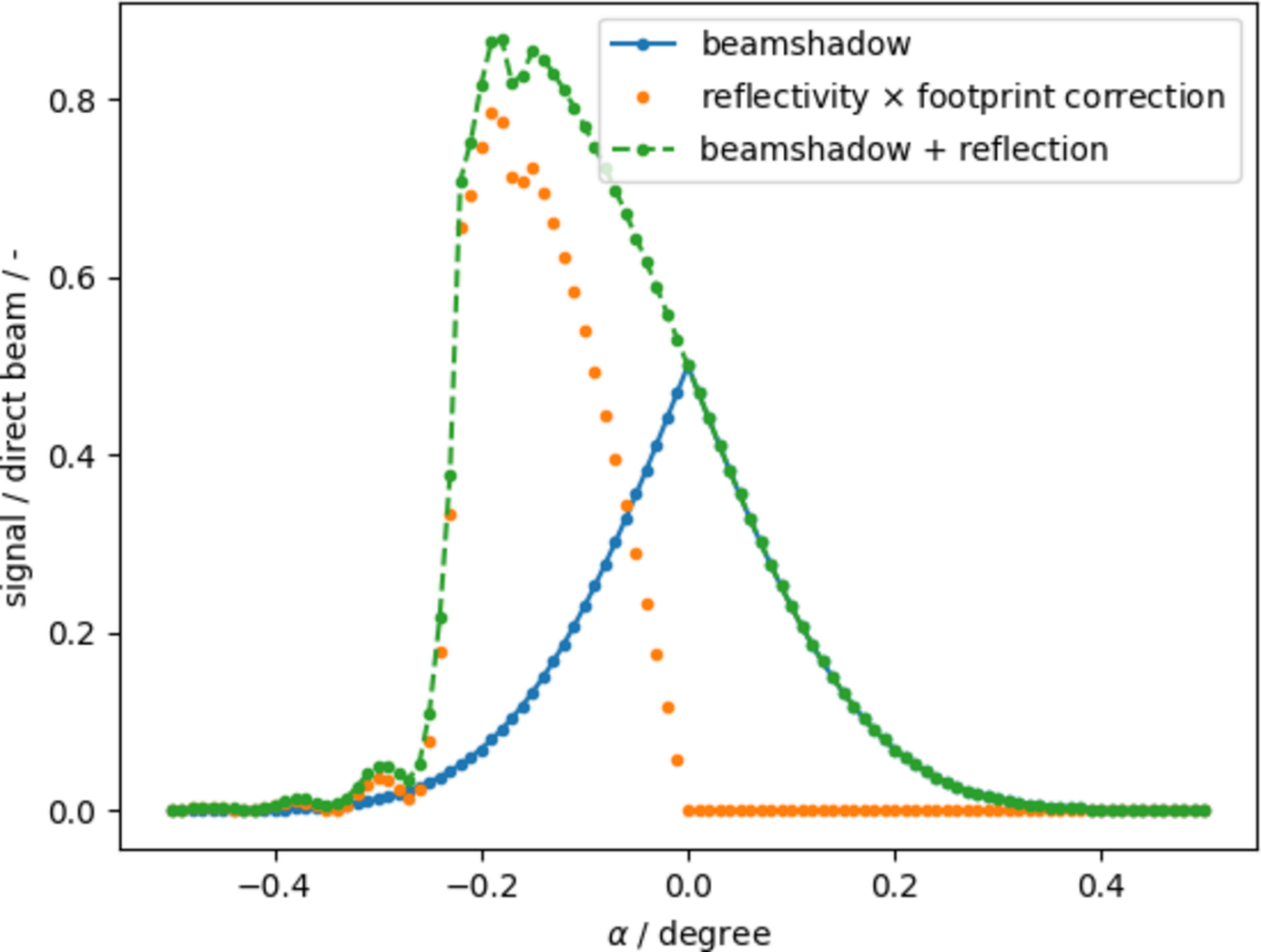
Signal recorded during incident angle scans. For the partially shadowed beam, the signal peaks at the sample’s horizontal position (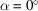), whereas the sum of the transmitted and reflected beam is maximal near the critical angle of the sample. A thin film of 50 nm of polystyrene on Si and Cu *K*α radiation was used to simulate the reflectivity.

Two main options exist to determine the relative sample orientation: using beam shadowing during coordinate scans (see *e.g.* Harrington & Santiso, 2021 ▸), and using the relative location of the specular reflection and direct beam. Depending on the type of radiation and flux, we will find different hardware that can separate the direct from the reflected beam (or not) and may be able to detect the location of the specular reflection. Our data on this question are summarized in Fig. 5 ▸. Only two respondents indicate no capability of separating the direct from the reflected beam. They have either the sum of the detector or a diode available and record the sum of the specular reflection and the partially obstructed beam during α (rocking) scans. The majority of answers (17) indicate that they can access the shadowed beam intensity separately, such that the signal 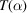 no longer depends on the sample’s reflectivity and peaks at incident angle zero (see Fig. 6 ▸). Eight instruments report reliance on the specular reflection for alignment purposes. In conclusion, respondents do not all have the same signal available for alignment purposes so their alignment routines necessarily differ. Conditional recommendations would therefore be the closest approximation to a reference method for sample alignment.

**Figure 7:**
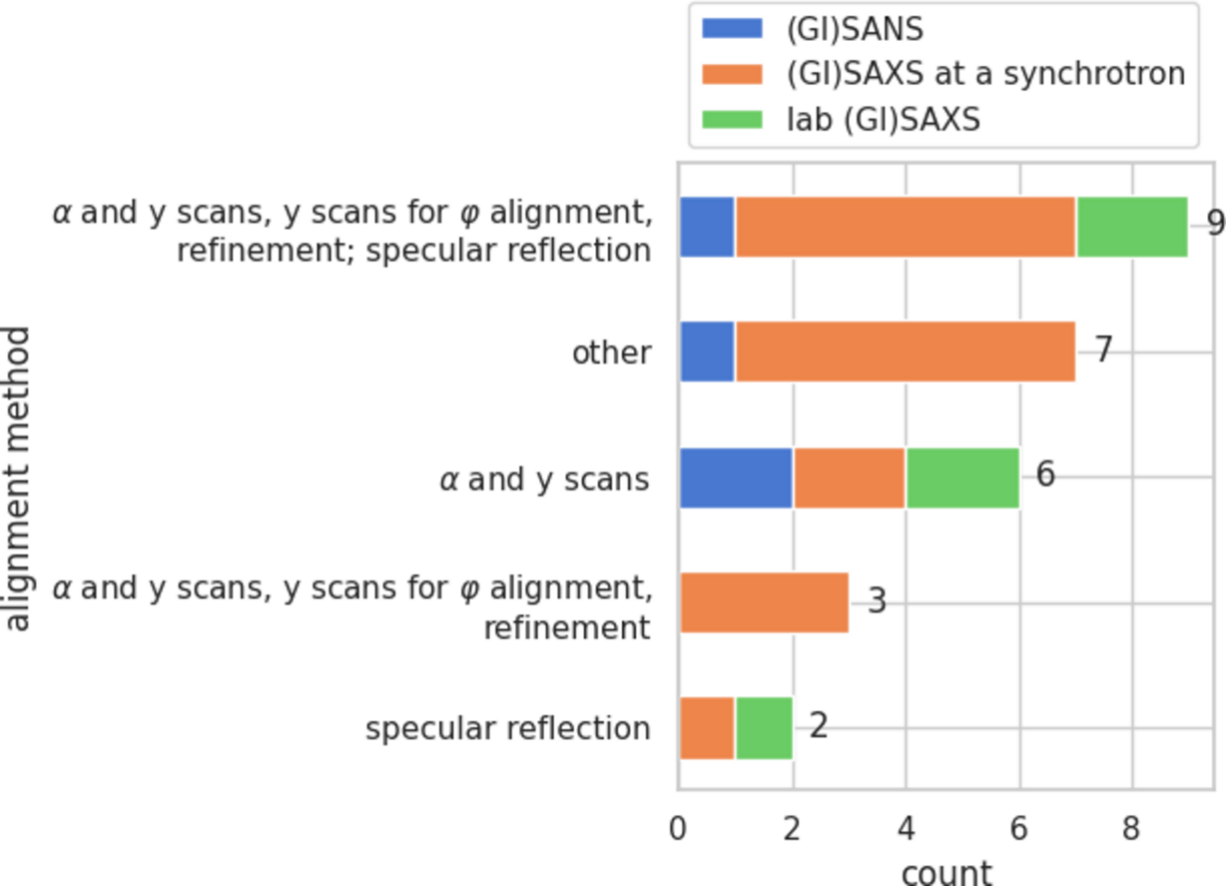
Unique alignment routine combinations and their occurrence in the dataset.

Fig. 7 ▸ shows the variability of responses to the question ‘What sequence of steps do you use to align individual samples?’. While leading with a few multiple-choice options, this question allowed free-form replies as well. Nine respondents combine methods using beam shadowing with those based on the specular reflection; another nine solely employ beam shadowing based methods. Seven respondents gave unique combinations of answers, of which five are free-form answers, as shown in the supporting information (Fig. S2). To summarize, the community has not converged on a sample alignment method. The differences do not appear to be due to the different sources of radiation in the dataset. Let us now see whether agreement is greater when it comes to calibration methods.

### 3.3. Instrument calibration

The term ‘instrument calibration’ refers to the process of determining the relative position of the detector with respect to the beam and the (aligned) sample. For any GI experiment, we require at least the sample-to-detector distance and the angle of incidence zero, which is determined during the sample alignment described in the previous section. Sample-to-detector distance calibration is in some ways more important than sample alignment: at least if the specular reflection can be detected during the measurement, we can retroactively determine the angle of incidence on the basis of the reflection location and a reliable calibration of the sample-to-detector distance. We use ‘calibration’ as shorthand for the calibration of the sample-to-detector distance and angle of incidence in this work but acknowledge that other quantities (*e.g.* resolution, detector tilt) may need to be calibrated as well.

**Figure 8:**
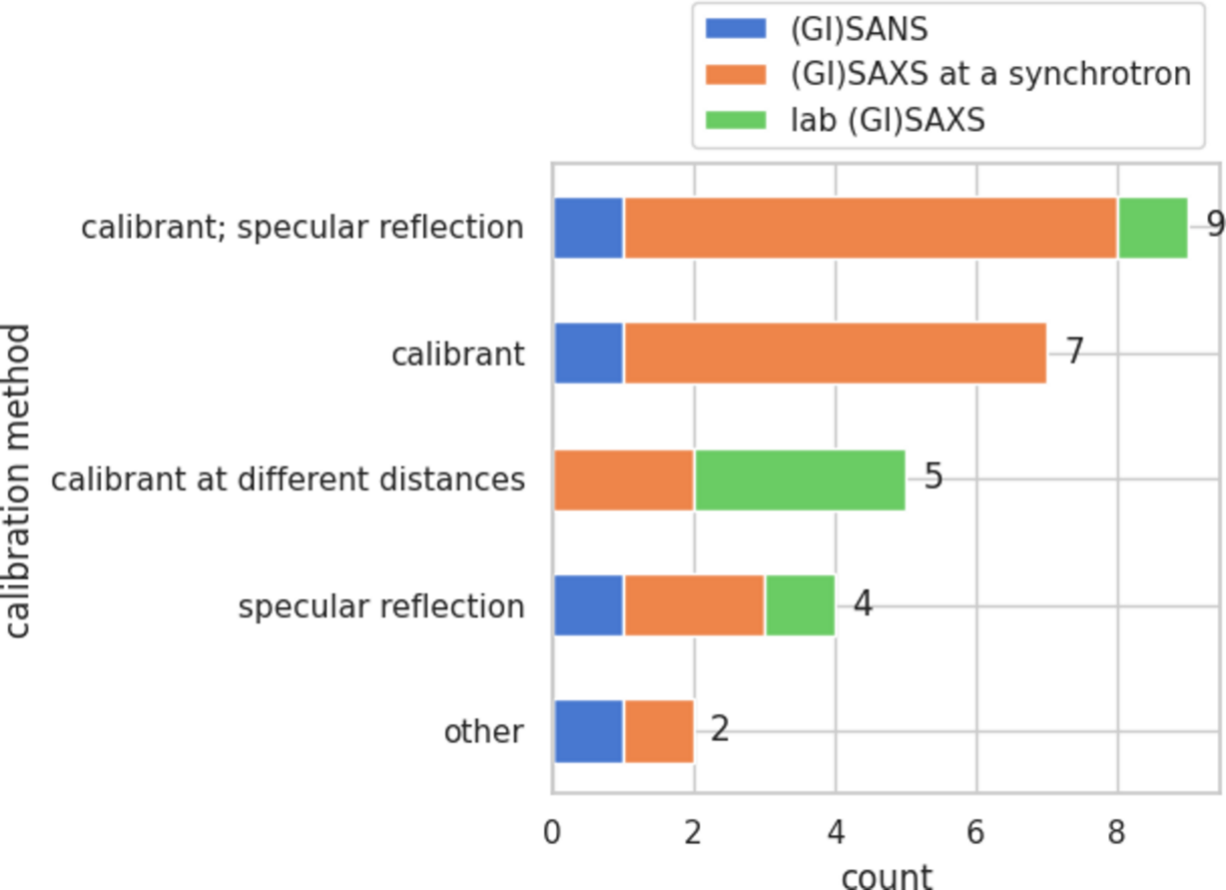
Unique combinations of signal(s) used to calibrate angle and sample-to-detector distance and their occurrence in the dataset.

Fig. 8 ▸ shows a relative majority in the responses for the combination of calibrant and specular reflection. The response overall is less diverse compared with that for the alignment method – it appears that fewer bespoke calibration methods have been developed. As proponents ourselves (Smales & Pauw, 2021 ▸) of the purely geometric triangulation method (Meli *et al.*, 2012 ▸; Wernecke *et al.*, 2014 ▸), we encourage the use of calibrants at different distances. The majority of users of this technique are laboratory instrument scientists (as stated above, our laboratory instrument is not in the dataset). Even if concrete choices such as the number of points used for calibration and whether calibration is done in transmission or reflection geometry (Lee *et al.*, 2006 ▸) are not reflected in the dataset, an agreement seems more tangible here. Once a recommended general principle for instrument calibration is identified, work on questions like uncertainties of the calibration results and subsequently on **q** for data corrections can begin.

## 4. Conclusions and outlook

In conclusion, there are no reference methods [in the technical sense of the term (Currie, 1995 ▸)] for GISAS alignment and calibration – or, at least, none that are used by the community. Community-driven actions are therefore the next logical step towards standardization, aided by data and contacts gathered in this work.

It appears that the community has partially converged in the three aspects studied here, hardware and software, sample alignment, and instrument calibration. We find two ‘camps’ with partial overlap in each of these areas: motor stages and hexapods for hardware, beam shadowing and use of the specular reflection for sample alignment, and use of a calibrant and/or use of the specular reflection for calibration. The responses reveal many home-cooked procedures. Unearthing this wealth of tips and tricks, for example the use of the Yoneda band for alignment purposes, is in line with the survey’s goals; however, we perceive a silent call for standardization therein as well. Mentions of manual steps (‘alignment by eye’) in particular call into question reproducibility, not only for alignment but also for the choice of ‘the best spot on the sample’ for the measurement. Measurement procedures are out of the scope of this work, though, as are many other aspects which could be prioritized, depending on the needs of the community.

Community needs and next actions were not included in the survey. We have queried ‘need for standardization’ on an ‘urgent and important’ to ‘neither urgent nor important’ scale (supporting information, Fig. S4) and find the vast majority in favor of standardization. To resolve what actions should be taken, follow-up questions were sent by e-mail. The most important needs and actions condensed from the replies are as follows:

(i) Information-type needs of scientists in charge of instruments:

(*a*) A quantitative and citeable evaluation of different alignment methods with uncertainty estimates. This is especially important for newcomers, *e.g.* new laboratory instruments.^5^

(*b*) Analysis of different hardware with respect to resolution and background. In particular, the angular alignment precision is needed for analysis until data reduction methods are perfected.

(ii) Networking-based development of the method:

(*a*) An agreement for naming angles and directions to facilitate discussions.

(*b*) An agreement on alignment procedures, in particular for highly reflecting, rough or partially absorbing surfaces.

(*c*) An agreement on data reduction and simulation methods, beginning with the exchange of knowledge.

(*d*) Round robin experiment(s), used as input for a ‘best practices’ document.

(iii) User management needs:

(*a*) Workshops with hands-on teaching from raw data to first analysis results.^6^

(*b*) A network of GI instruments at large-scale facilities as a single point of access for users and to encourage instrument complementarity.

Fulfillment of these needs fosters good scientific practice, enables deduplication of efforts and is preparation for standardization. However, this requires continued effort by scientists on the fundamentals – not a trivial matter in today’s research environment.

Next actions matching the above needs are strengthening connections in the group formed on the basis of the survey (the Grazing Ray Initiative, https://github.com/grazingrays/), establishing good contact with neighboring efforts like the Open Reflectometry Standards Organization and other canSAS working groups, monitoring the field for standardization-related work [*e.g.* Abukaev *et al.*’s (2026 ▸) data format], and knowledge exchange on subtopics like sample alignment.

GI scattering has been around for decades now, without indications of decline. To fully establish the method alongside and between reflectometry and small-angle scattering, however, its boundaries as well as experimental methods require more definition.

## Supplementary Material

Supporting information (pdf of survey questions, countries where participating institutions are located, which techniques are mentioned by our survey participants, lists of synchrotron and laboratory instruments, publication activity in the field, unique alignment routines, and most frequently mentioned software). DOI: 10.1107/S1600576726005741/jl5136sup1.pdf

Survey response data: https://doi.org/10.5281/zenodo.18712813

Literate programming source of this work: https://doi.org/10.5281/zenodo.18713631

## Figures and Tables

**Table 1:** Number of instruments by source of radiation

Type	Count
(GI)SAXS at a synchrotron	18
Laboratory (GI)SAXS	5
(GI)SANS	4

**Table 2:** Notation conventions used in this work, following the NeXuS coordinate system definition

Symbol	Field name	Type	Vector
ϑ	Polar angle	Rotation	0 1 0
φ	Azimuthal angle	Rotation	0 0 1
α	Meridional angle	Rotation	1 0 0
*z*	Distance	Translation	0 0 1
*y*	Height	Translation	0 1 0
*x*	*x* translation	Translation	1 0 0

## Data Availability

The response data are available at https://doi.org/10.5281/zenodo.18712813. The literate programming source of this work is available at https://doi.org/10.5281/zenodo.18713631.

## Footnotes

1 Manual count: D33, Super-Adam, KWS-1, KWS-2, KWS-3, REFSANS, vSANS, MAGREF, QUOKKA, SANS-Suanni, SKADI.

2 Manual best-effort count based on instrument websites, see supporting information, Table S5.

3 Manual best-effort count based on instrument websites, see supporting information, Table S6.

4 Query example for GISAXS: https://openalex.org/works?page=1&filter=title_and_abstract.search:GISAXS,publication_year:2020-2025&view=list,report,api.

5 According to Xenocs, they have sold GISAXS equipment with nearly every instrument in recent years (personal communication).

6 Compare past GISAS schools in Bayreuth/Munich and GISAXS workshops in Hamburg, Germany (IUCr Commission on Small-Angle Scattering, 2026 ▸).

## Appendix A Notation

We use NeXuS coordinates. The symbols we employ to abbreviate the field names are given in Table 2 ▸.

## References

[bb2] Abukaev, A., Völter, C., Romodin, M., Schwartzkopff, S., Bertram, F., Konovalov, O., Hinderhofer, A., Lapkin, D. & Schreiber, F. (2026). *J. Appl. Cryst.* **59**, 263–275. 10.1107/S1600576725010593 PMC12871486 41647184

[bb3] Blanton, T. N., Barnes, C. L. & Lelental, M. (2000). *J. Appl. Cryst.* **33**, 172–173.

[bb4] Currie, L. A. (1995). *Pure Appl. Chem.* **67**, 1699–1723.

[bb5] Dacombe, M. (2017). *Acta Cryst.* A**73**, 159–199. 10.1107/S2053273316011128 28248666

[bb6] Daillant, J. & Gibaud, A. (2009). Editors. *X-ray and Neutron Reflectivity.* Lecture Notes in Physics. Berlin: Springer.

[bb7] DeLongchamp, D. M., Kline, R. J., Fischer, D. A., Richter, L. J. & Toney, M. F. (2011). *Adv. Mater.* **23**, 319–337. 10.1002/adma.201001760 20809510

[bb8] Frömsdorf, A., Čapek, R. & Roth, S. V. (2006). *J. Phys. Chem. B* **110**, 15166–15171. 10.1021/jp0621880 16884231

[bb9] Harrington, G. F. & Santiso, J. (2021). *J. Electroceram.* **47**, 141–163.

[bb10] Hexemer, A. & Müller-Buschbaum, P. (2015). *IUCrJ* **2**, 106–125. 10.1107/S2052252514024178 PMC4285885 25610632

[bb11] Holzer, V., Schrode, B., Simbrunner, J., Hofer, S., Barba, L., Resel, R. & Werzer, O. (2022). *Rev. Sci. Instrum.* **93**, 063906. 10.1063/5.0088176 35778026

[bb1] International Organization for Standardization (2020). Standard ISO 16413:2020(E). International Organization for Standardization, Geneva, Switzerland. https://www.iso.org/standard/76403.html.

[bb12] IUCr Commission on Small-Angle Scattering (2026). *All events*, https://www.iucr.org/resources/commissions/small-angle-scattering/all-events.

[bb13] Köhler, S., Arnold, T., Birch, J., Cárdenas, M., Dorri, S., Månsson, M., Nylander, T., Rogers, S., Roth, S. V., Sassa, Y. & Wolff, M. (2026). *Adv. Colloid Interface Sci.* **349**, 103757. 10.1016/j.cis.2025.103757 41422768

[bb14] Kuhn, M., Huang, X., Gebert, M., Thomsen, L., McNeill, C. R. & Herzig, E. M. (2025). *Adv. Funct. Mater.* **35**, 2509532.

[bb15] Lee, B., Lo, C.-T., Seifert, S. & Winans, R. E. (2006). *J. Appl. Cryst.* **39**, 749–751.

[bb16] Meli, F., Klein, T., Buhr, E., Frase, C. G., Gleber, G., Krumrey, M., Duta, A., Duta, S., Korpelainen, V., Bellotti, R., Picotto, G. B., Boyd, R. D. & Cuenat, A. (2012). *Meas. Sci. Technol.* **23**, 125005.

[bb17] Pospelov, G., Van Herck, W., Burle, J., Carmona Loaiza, J. M., Durniak, C., Fisher, J. M., Ganeva, M., Yurov, D. & Wuttke, J. (2020). *J. Appl. Cryst.* **53**, 262–276. 10.1107/S1600576719016789 PMC6998781 32047414

[bb18] Priem, J., Piwowar, H. & Orr, R. (2022). *arXiv*, 2205.01833.

[bb19] Renaud, G., Lazzari, R. & Leroy, F. (2009). *Surf. Sci. Rep.* **64**, 255–380.

[bb20] Reus, M. A., Reb, L. K., Kosbahn, D. P., Roth, S. V. & Müller-Buschbaum, P. (2024). *J. Appl. Cryst.* **57**, 509–528. 10.1107/S1600576723011159 PMC11001412 38596722

[bb21] Smales, G. J. & Pauw, B. R. (2021). *J. Instrum.* **16**, P06034.

[bb22] Smilgies, D. (2021). *J. Polym. Sci.* **60**, 1023–1041.

[bb23] Smilgies, D.-M. (2025). *Crystals* **15**, 63.

[bb24] Steele, J. A., Solano, E., Hardy, D., Dayton, D., Ladd, D., White, K., Chen, P., Hou, J., Huang, H., Saha, R. A., Wang, L., Gao, F., Hofkens, J., Roeffaers, M. B. J., Chernyshov, D. & Toney, M. F. (2023). *Adv. Energy Mater.* **13**, 2300760.

[bb25] Thompson, A. C. (2009). Editor. *X-ray Data Booklet.* Lawrence Berkeley Laboratory, University of California, USA.

[bb26] Tortorici, E. & Rogers, C. T. (2025). *arXiv*, 2506.22970.

[bb27] Vegso, K., Siffalovic, P., Benkovicova, M., Jergel, M., Luby, S., Majkova, E., Capek, I., Kocsis, T., Perlich, J. & Roth, S. V. (2012). *Nanotechnology* **23**, 045704. 10.1088/0957-4484/23/4/045704 22222583

[bb28] Vineyard, G. H. (1982). *Phys. Rev. B* **26**, 4146–4159.

[bb29] Wernecke, J., Krumrey, M., Hoell, A., Kline, R. J., Liu, H.-K. & Wu, W.-L. (2014). *J. Appl. Cryst.* **47**, 1912–1920.

[bb30] Wernecke, J., Scholze, F. & Krumrey, M. (2012). *Rev. Sci. Instrum.* **83**, 103906. 10.1063/1.4758283 23126781

[bb31] Werzer, O., Kowarik, S., Gasser, F., Jiang, Z., Strzalka, J., Nicklin, C. & Resel, R. (2024). *Nat. Rev. Methods Primers* **4**, 15.

