# Supplementary material for: Grazing-incidence scattering surveyed: towards reference methods for alignment and calibration
Source: J Appl Crystallogr. 2026 Jul 24;59(Pt 4):1247–53. doi: 10.1107/S1600576726005741 (PMC13440042; doi:10.1107/S1600576726005741)
Supplement: Supplementary file 1 [file j-59-01247-sup1.pdf]

JOURNAL OF
APPLIED
CRYSTALLOGRAPHY

Volume 59 (2026)

Supporting information for article:

Grazing-incidence scattering surveyed: towards reference methods for alignment and calibration

Anja F. Hörmann, Daniel M. Balazs, Ingo Breßler, Sumea Klokic, Melika Moradi, Eduardo Solano, Annika Stellhorn and Brian R. Pauw

Supporting information

Grazing incidence scattering surveyed: toward reference methods for alignment and calibration

Anja F. Hörmann ✉ [a], Daniel M. Balazs [b], Ingo Breßler [a], Sumea Klokic [c], Melika Moradi [a], Eduardo Solano [d], Annika Stellhorn [e], and Brian R. Pauw [a]

[a]Bundesanstalt für Materialforschung und -prüfung, Unter den Eichen 87, 12205 Berlin, Germany
[b]Institute of Science and Technology Austria, Am Campus 1, 3400 Klosterneuburg, Austria
[c]Graz University of Technology, Stremayrgasse 9, 8010 Graz, Austria
[d]ALBA Synchrotron, Carrer de la Llum 2-26, 08290 Cerdanyola del Vallès, Barcelona, Spain
[e]European Spallation Source ERIC, Box 176, SE-221 00, Lund, Sweden

July 27, 2026

We report

- survey questions (table 1),
- countries where participating institutions are located (table 2),
- which techniques are mentioned by our survey participants (table 3),
- our best-effort lists of synchrotron (table 5) and laboratory instruments (table 6) with grazing incidence capabilities,
- publication activity (table 4),
- unique alignment routines (Fig. 2) and
- most frequently mentioned software (Fig. 3).

List of Tables

3 Grazing incidence techniques mentioned in survey responses or on linked websites. 2
5 Synchrotron beamlines with self-reported GI capabilities. 3
1 Survey questions 6
2 Location of participating institutions 6

4 Publication numbers according to three search engines and two date ranges when querying for four grazing incidence methods as their acronyms. Search queries were limited to title and abstract of the works (Openalex), title, abstract and keyword (Scopus, Web of Science). With the exception of GISANS, where Openalex reports significantly higher numbers, data from all three search engines are consistent. 7
6 Laboratory instruments with self-reported GI capabilities 10

List of Figures

1 Publication numbers according to three search engines by year for four different technique acronyms. Search queries were limited to title and abstract of the works (Openalex), title, abstract and keyword (Scopus, Web of Science). With the exception of GISANS, where Openalex reports significantly higher numbers, data from all three search engines are consistent. 7
2 Unique alignment routines and their occurrence in the dataset. 8
3 Most frequently mentioned software/frameworks available to control the instrument. The data represents a snapshot, several respondents mentioned phasing out spec. Multiple answers were allowed for this multiple-choice question. 8
4 Answers to the question ”How great a need do you see for standardization in GISAS?” on a scale from ”neither urgent nor important” to ”urgent and important”. A clear majority gave answers in favour of standardisation, but we expect sampling bias toward people with an interest in standardisation. 9

Table 3: Grazing incidence techniques mentioned in survey responses or on linked websites.

	institution	link	GI keywords
0	Advanced Light Souce LBNL	7.3.3	GISAXS
1	Advanced Photon Source, Argonne National Lab	9-ID-D GI-SAXS/WAXS/XPCS	GISAXS, GIWAXS
2	BL15A PF KEK Japan	BL-15A2	GISAXS
3	ESRF	BM26 (DUBBLE-CRG)	GISAXS, GIWAXS
4	ANSTO	Bilby	GISANS
5	Canadian Light Source	Brockhouse Sector Wiggler Low Energy (BXDS)	GISAXS
6	National Synchrotron Light Source II	Complex Materials Scattering (CMS)	GISAXS, GIWAXS
7	BESSY II / PTB	FCM beamline	GISAXS

Continued on next page

Table 3: Grazing incidence techniques mentioned in survey responses or on linked websites.

	institution	link	GI keywords
8	Advanced Photon Source (APS-U), Argonne National Laboratory	11-ID-B Total x-ray scattering	GI Diffraction, GITXS, GISAXS
9	Laboratoire Léon Brillouin, CEA Saclay	news: moving to JNP	GISANS
10	Diamond Light Source	I22	GISAXS, GIWAXS
11	ESRF	ID31	GISAXS
12	ALBA synchrotron	NCD-SWEET	GISAXS, GIWAXS
13	PETRA III (DESY, Hamburg)	P03 - MiNaXS	GISAXS, GIWAXS
14	PETRA III	P07-EH2 & P21.1	grazing incidence diffraction, grazing incidence total scattering
15	Synchrotron	SAXS beamline -> HF-SAXS	GISAXS
16	Graz University of Technology	TU Graz	GISAXS
17	Anton Paar	SAXS/WAXS-System: SAXSpoint 500	GISAXS, GIWAXS
18	ESS	SKADI	GISANS
19	National Synchrotron Light Source II	SMI (OPLS endstation)	GISAXS, GIWAXS
20	National Synchrotron Light Source II	SMI (SAXS/WAXS endstation)	GISAXS, GIWAXS
21	ISIS	SANS2D sample environments	GISANS
22	synchrotron SOLEIL	SixS	GISAXS
23	ESRF	XMaS BM28	grazing incidence diffraction, GISAXS, GIWAXS
24	University of Colorado	University of Colorado (institute)	GISAXS, GIWAXS
25	Diamond Light Source	labSAXS	GISAXS
26	Institute of Science and Technology Austria	BMFWF	GISAXS

Table 1: Survey questions

#	question
1	What large scale facility or other organization is your instrument located at?
2	What is the name of your instrument?
3	What type of instrument is it?
4	What characteristic sets your instrument apart from all or most others?
5	What type of hardware allows you to control the relative positions and orientations of beam and sample?
6	Which degrees of freedom can you control specifically?*
7	What sample orientations do you use on a regular basis?
8	What option(s) do you have to measure the direct beam?
9	What option(s) do you have to measure the specularly reflected beam?
10	What do you use as a sample holder?
11	If you wanted to change a procedure at your instrument, e.g. a sequence of scans for alignment, how is that possible?
12	What interface(s) do you have to control your instrument?
13	What measures are available to quantify shadowing of the beam?
14	What sequence of steps do you use to align individual samples?
15	Is there an aspect of sample alignment you payed particular attention to? Something you might do differently than anybody else?
16	Please share your alignment routine (or representative section thereof) via - link to a public repo or - by contributing to this gist: https://gist.github.com/ahoermann/d44605100109964ccdbd2f...
17	What signal do you use for angle and sample-to-detector calibration?
18	Is there an aspect of calibration you payed particular attention to? Something you might do differently than anybody else?
19	Please share your calibration routine (or representative section thereof) via - link to a public repo or - by contributing to this gist: https://gist.github.com/ahoermann/d44605100109964ccdbd...
20	Would you like to participate in this standardization project beyond participation in this survey?
21	How great a need do you see for standardization in GISAS?
22	Have we overlooked anything? Is there a resource you'd like to point us to?

Table 2: Location of participating institutions

country	count
USA	7
France	5
Austria	3
UK	3
Germany	3
Canada	1
Spain	1
Australia	1
Italy	1
Japan	1
Sweden	1

Table 4: Publication numbers according to three search engines and two date ranges when querying for four grazing incidence methods as their acronyms. Search queries were limited to title and abstract of the works (Openalex), title, abstract and keyword (Scopus, Web of Science). With the exception of GISANS, where Openalex reports significantly higher numbers, data from all three search engines are consistent.

	Openalex		Scopus		Web of Science	
	2020-2025	1991-2025	2020-2025	1991-2025	2020-2025	1991-2025
GISAXS	325	1658	307	1436	325	1434
GIWAXS	572	965	565	933	569	925
GISANS	48	179	30	112	31	108
GIXRD	758	2452	849	2446	799	2265
sum	1703	5254	1751	4927	1724	4732

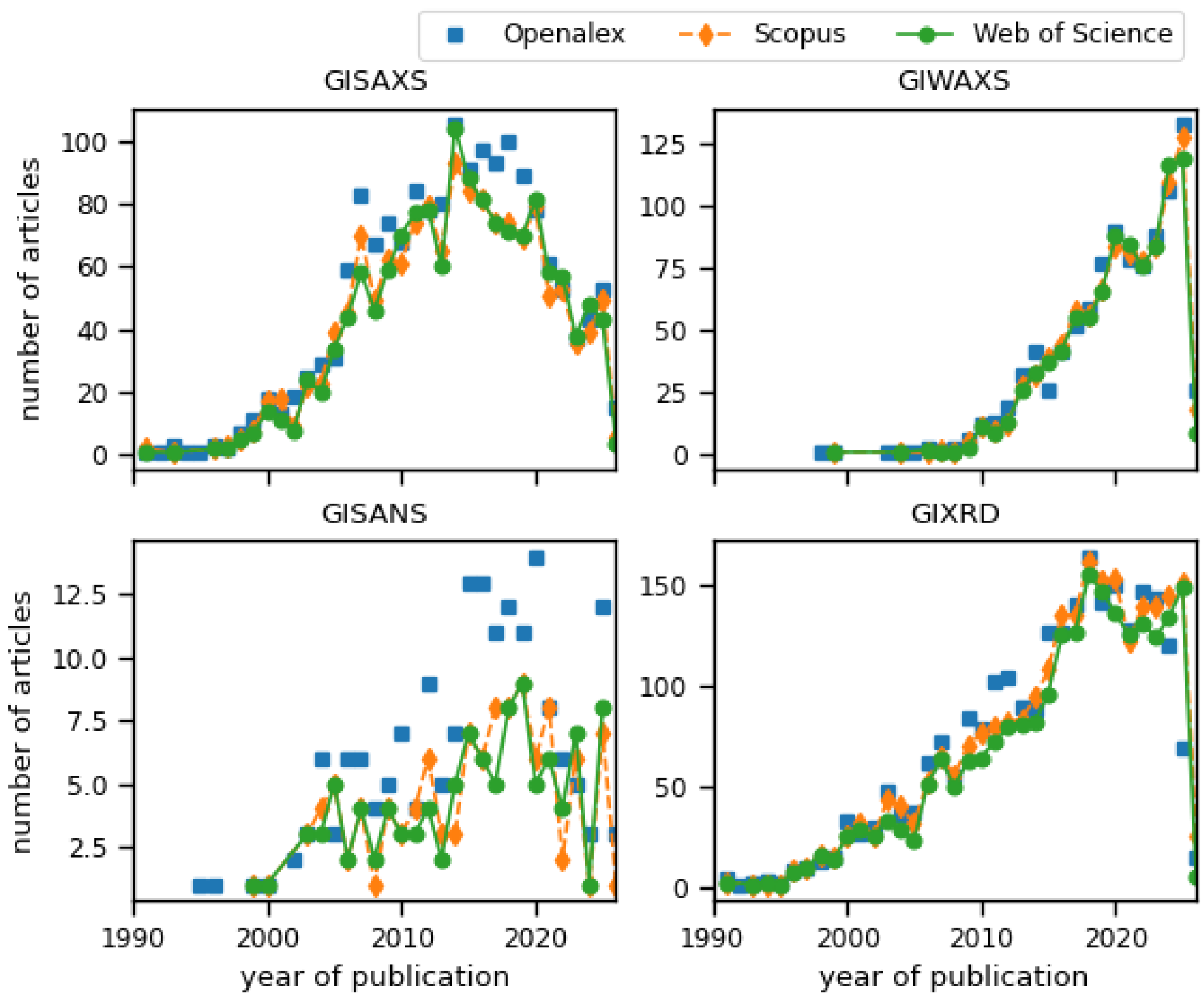

Figure 1: Publication numbers according to three search engines by year for four different technique acronyms. Search queries were limited to title and abstract of the works (Openalex), title, abstract and keyword (Scopus, Web of Science). With the exception of GISANS, where Openalex reports significantly higher numbers, data from all three search engines are consistent.

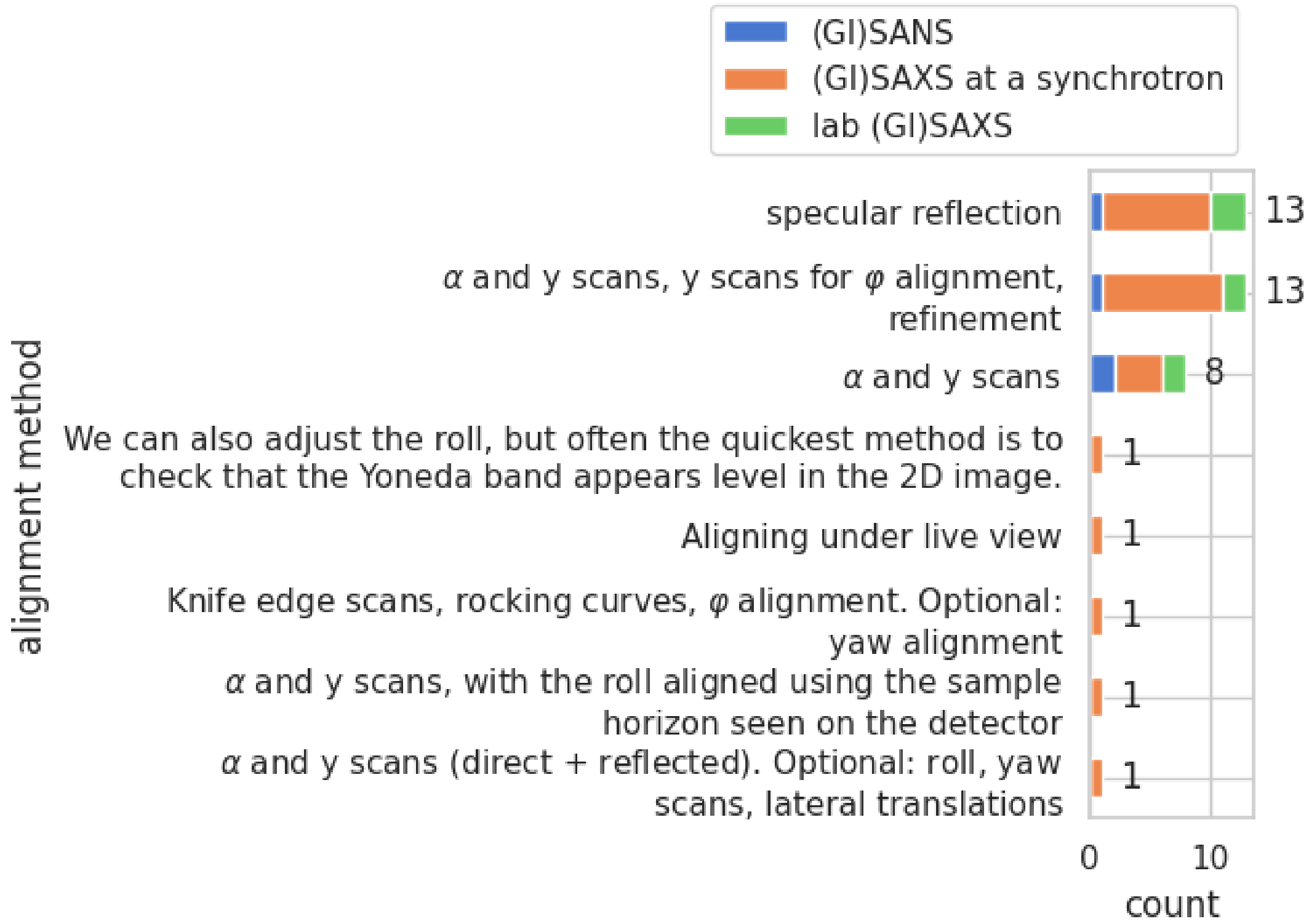

Figure 2: Unique alignment routines and their occurrence in the dataset.

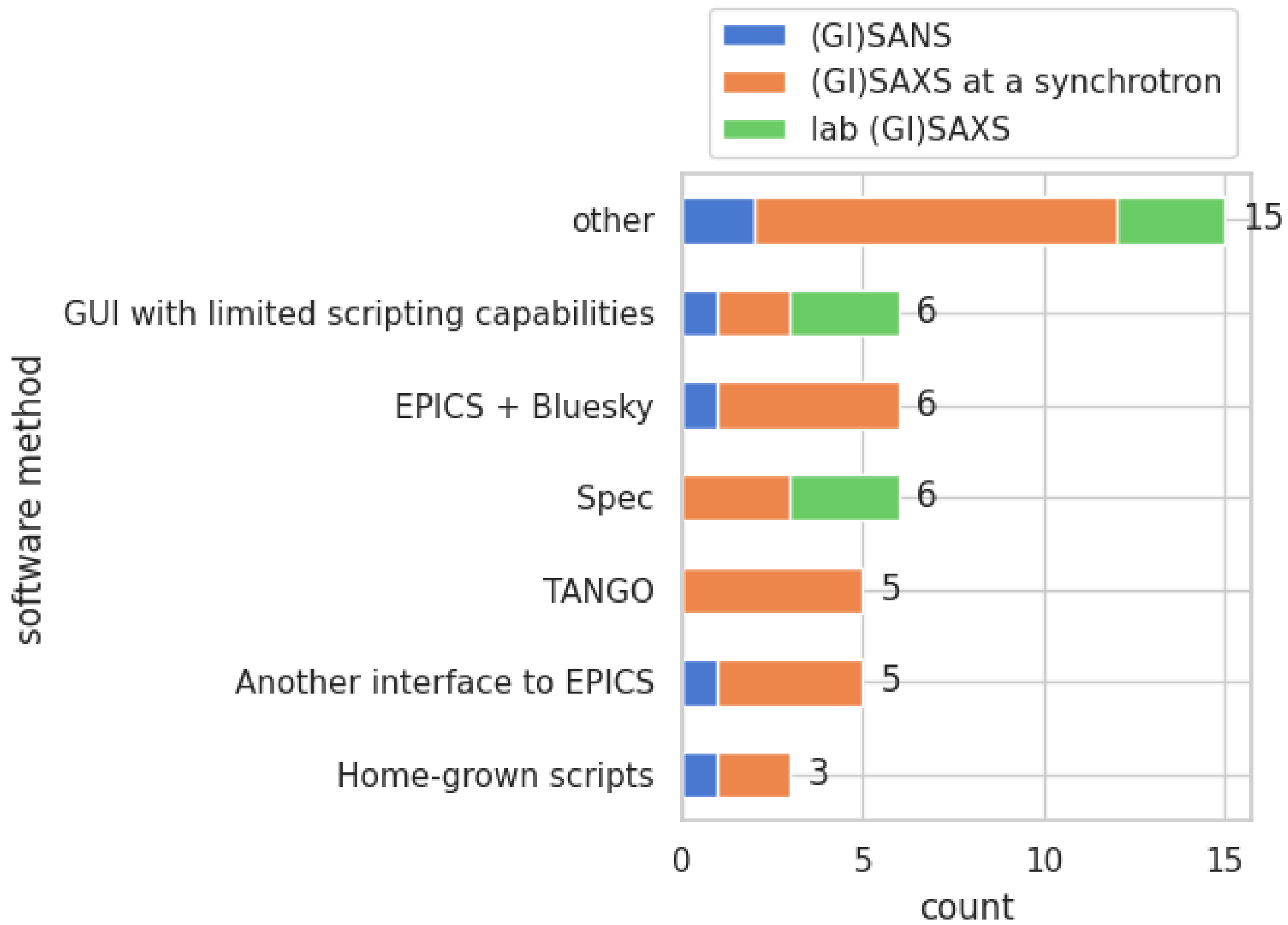

Figure 3: Most frequently mentioned software/frameworks available to control the instrument. The data represents a snapshot, several respondents mentioned phasing out spec. Multiple answers were allowed for this multiple-choice question.

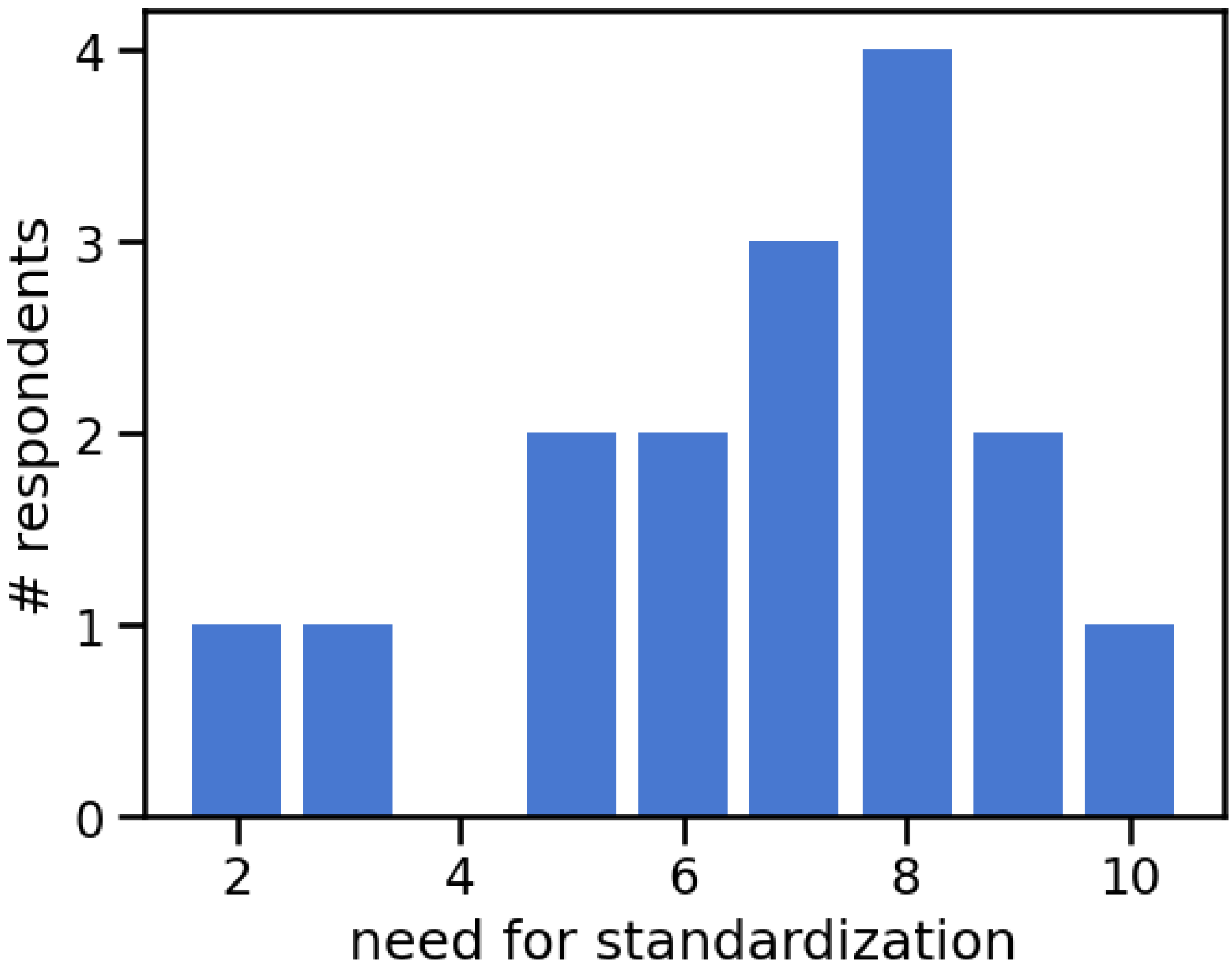

Figure 4: Answers to the question "How great a need do you see for standardization in GISAS?" on a scale from "neither urgent nor important" to "urgent and important". A clear majority gave answers in favour of standardisation, but we expect sampling bias toward people with an interest in standardisation.

Table 5: Synchrotron beamlines with self-reported GI capabilities.

	Country	Synchrotron	Beamline	GISAXS	GIWAXS
0	Australia	ANSTO	SAXS/WAXS beamline	True	False
1	Brazil	SIRIUS	JATOBÁ	True	True
2	Canada	Canadian LS	Brockhouse Sector Wiggler Low Energy (BXDS)	True	False
3	China	BSRF	1W2A	True	False
4	China	SSRF	BL16B1	True	True
5	France	ESRF	ID01	True	False
6	France	ESRF	ID31	True	False
7	France	ESRF	BM02	True	False
8	France	ESRF	XMaS BM28	True	True
9	France	ESRF	BM32	True	False
10	France	ESRF	BM26 (DUBBLE-CRG)	True	True
11	France	ESRF	ID10	True	True
12	France	ESRF	ID13	True	False
13	France	SOLEIL	SixS	True	False
14	France	Soleil	SWING	True	False
15	Germany	BESSY II	FCM beamline	True	False
16	Germany	KIT light source	NANO beamline	True	False
17	Germany	PETRA III	P03 - MiNaXS	True	True
18	Germany	PETRA III	P62	True	False
19	Italy	ELETTRA 2.0	HF-SAXS	True	False
20	Italy	ELETTRA 2.0	HB-SAXS	True	True
21	Japan	Photon Factory (PF), KEK	BL-6A	True	True
22	Japan	Photon Factory (PF), KEK	BL-10C	True	True
23	Japan	Photon Factory (PF), KEK	BL-15A2	True	True
24	Japan	Spring-8	BL03XU	True	False
25	Japan	Spring-8	BL13XU	True	True
26	South Korea	Pohang Light Source-II (PLS-II)	3C	True	True

Continued on next page

Table 5: Synchrotron beamlines with self-reported GI capabilities.

	Country	Synchrotron	Beamline	GISAXS	GIWAXS
27	South Korea	Pohang Light Source-II (PLS-II)	9A	True	True
28	Spain	ALBA	NCD-SWEET	True	True
29	Sweden	MAX IV	coSAXS	True	True
30	Taiwan	TLS	BL23A1 → 14A	True	False
31	Taiwan	TLS	BL01C2 → 19A/20A	False	True
32	Taiwan	TPS	BL25A	True	False
33	UK	Diamond	I22	True	True
34	UK	Diamond	I07	True	False
35	USA	ALS	7.3.3	True	False
36	USA	ALS	11.0.1.2	True	True
37	USA	ALS	11.0.2	True	True
38	USA	APS	9-ID-D GI-SAXS/WAXS/XPCS	True	True
39	USA	APS	12-ID-E	True	False
40	USA	CHESS	MSN-C	True	True
41	USA	NSLS-II	SMI (SAXS/WAXS endstation)	True	True
42	USA	NSLS-II	SMI (OPLS end-station)	True	True
43	USA	NSLS-II	Complex Materials Scattering (CMS)	True	True

Table 6: Laboratory instruments with self-reported GI capabilities

	Country	Laboratory / Facility	Instrument (model)	Link	GI	survey
0	Argentina	INIFTA SAXS Lab (CONICET / UNLP)	Xenocs Xeuss	Facility link	Y	
1	Australia	The University of Sydney	SAXSPoint, Anton Paar	Facility link	Y	
2	Australia	University of Queensland	Xenocs Xeuss 2.0	Facility link	Y	
3	Austria	Anton Paar	SAXSpoint 5.0	SAXS/WAXS-System: SAXSpoint 500	Y	Y
4	Austria	BOKU Wien	PSAXS System S/Max 3000 RIGAKU	BOKU	Y	
5	Austria	Institute of Science and Technology Austria	Xenocs Xeuss 3.0	BMFWF	Y	Y
6	Austria	SOMAPP Lab (TU Graz)	Anton Paar SAXSpoint 2.0	TU Graz	Y	Y
7	Austria	UniLeoben	Nanostar, Bruker AXS	Equipment	Y	
8	Austria	Universität SALZBURG	Xenocs Xeuss 3.0	Universität Salzburg	Y	
9	Brazil	CNANO-UFRGS — Center for Nanoscience	Xenocs Nano-inXider	UFRGS CNANO	Y	

Continued on next page

Table 6: Laboratory instruments with self-reported GI capabilities

	Country	Laboratory / Facility	Instrument (model)	Link	GI	survey
10	Czech Republic	MGML (Materials Growth & Measurement Lab)	Xenocs Xeuss 2.0	Facility link	Y	
11	Denmark	University of Copenhagen	Customized SAXS	CXC: Cross-disciplinary X-ray Center	Y	
12	France	SWAXS Lab — LLB / IRAMIS (CEA Saclay)	Xenocs Xeuss 2.0	LLB Facility	Y	
13	Germany	Bundesanstalt für Materialforschung und -prüfung (BAM)	MOUSE / modified Xenocs Xeuss 2.0	Blog post on grazing incidence survey	Y	
14	Germany	Erlangen Scattering Methods Lab (FAU Erlangen)	Xenocs VAXSTER & SAXSpace	CENEM Lab	Y	
15	Germany	MLZ SAXS Lab VAXTER (FRM II, Garching)	Customized Xenocs SAXS/WAXS	MLZ site	Y	
16	India	CRF Sonipat Campus	Anton Paar SAXSpoint 2.0	CRF	Y	
17	India	IIT Kanpur	custom, microsource	SAXS Lab	Y	

Continued on next page

Table 6: Laboratory instruments with self-reported GI capabilities

	Country	Laboratory / Facility	Instrument (model)	Link	GI	survey
18	India	Indian Institute of Technology Kharagpur	Xenocs	SAXS Lab	Y	
19	India	Panjab University	no information	SAXS Lab	Y	
20	Italy	SAXSlab (Sapienza University of Rome)	Xenocs Xeuss 2.0 HR	SAXSlab	Y	
21	Norway	UiO (University of Oslo) - RECX	Bruker Nanostar	Small Angle Scattering	Y	
22	Poland	Zakład Fizyki Biomedycznej SAXS Lab (Adam Mickiewicz Univ.)	Xenocs Xeuss 2.0	Facility link	Y	
23	Russia	Research Center for Molecular Mechanisms	Rigaku	Facility link	Y	
24	Singapur	Nanyang Technological University	SWAXS Anton Paar SAXSess	SAXS	Y	
25	Slovakia	Institute of Physics, Slovak Academy of Sciences	various, customized	Laboratory of Multilayers and Nanostructures	Y	
26	Spain	University of Valencia	Bruker N8 HORIZON	SAXS	Y	

Continued on next page

Table 6: Laboratory instruments with self-reported GI capabilities

	Country	Laboratory / Facility	Instrument (model)	Link	GI	survey
27	Sweden	RISE SAXS Service Lab	Anton Paar SAXSpoint	RISE	Y	
28	Sweden	SAXS:Mat:Nordic	customized ?	CHALMERS	Y	
29	Switzerland	EMPA Laboratory	Bruker Nanostar	link	Y	
30	Switzerland	EMPA Laboratory	Bruker D8	link	Y	
31	UK	Diamond labSAXS	Xenocs Xeuss 3.0	Diamond	Y	
32	UK	Soft Matter Analytical Lab (University of Sheffield)	Xenocs Xeuss 2.0; Bruker Nanostar	Facility link	Y	
33	UK	University of Liverpool	Bruker Nanostar system	ROYCE	Y	
34	USA	BNL CFN Labscale GISAXS setup (CFN/BNL)	Custom lab instrument (Bruker/SAXSLAB)	Facility link	Y	
35	USA	BioPACIFIC MIP SAXS/WAXS (UC Santa Barbara / UC NanoSystems Institute)	Next-gen SAXS/WAXS system with GISAXS	Facility link	Y	
36	USA	CINT	SmartLab RIGAKU	CINT	Y	
37	USA	CINT	Xenocs Xeuss 3.0	CINT	Y	

Continued on next page

Table 6: Laboratory instruments with self-reported GI capabilities

	Country	Laboratory / Facility	Instrument (model)	Link	GI	survey
38	USA	CapNetEq	Xenocs Xeuss 3.0	Facility link	Y	
39	USA	Eyring Materials Center (Arizona State University)	Rigaku SmartLab	ASU Eyring	Y	
40	USA	Louisiana State University/CAMD	beamline or laboratory instrument?	CAMD	Y	
41	USA	MIT SAXS-LAB Retro-F	Customized	MIT.nano	Y	
42	USA	McGill University	Anton Paar SAXSpoint 2.0	McGill Characterization Facility	Y	
43	USA	Oak Ridge National Laboratory	Xenocs Xeuss 3.0	Oak Ridge	Y	
44	USA	Texas A&M University Materials Center	Xenocs Xeuss 3.0	TAMU Core	Y	
45	USA	UC San Diego MR-SEC	Xenocs Xeuss 3.0	lab	Y	
46	USA	University of Chicago	Xenocs	University Chicago	Y	
47	USA	University of Colorado	Xenocs		Y	Y

Continued on next page

Table 6: Laboratory instruments with self-reported GI capabilities

	Country	Laboratory / Facility	Instrument (model)	Link	GI	survey
48	USA	University of Illinois Urbana-Champaign	Genix3D ULD microfocus source/costum lab	Materials Research Laboratory	Y	
49	USA	University of Maryland XRCF	Xenocs Xeuss SAXS/WAXS/GISAXS	UMD XRCF	Y	
50	USA	University of Nebraska–Lincoln	Anton Paar SAXSpoint 5.0	UNL Core Facility	Y	
51	USA	University of South Carolina	Customized, GeniX 3D microfocus	SAXS Lab	Y	
52	USA	University of Southern Mississippi	Xenocs Xeuss 2.0	SPSE Scattering	Y	
53	USA	University of Tennessee	Xenocs Xeuss 3.0	The PCL	Y	
54	USA	University of Utah Laboratory Beamline	Anton Paar SAXSpoint 5.0	EM and Surface analysis lab	Y	
55	USA	University of Warwick	Xenocs Xeuss 2.0	Lab	Y	
56	USA	Washington State University, Nuclear Science Center	Xenocs Xeuss 3.0	Spectroscopy lab	Y	
